# The Landsteiner lung cancer research platform (LALUCA): Objectives, methodology and implementation of a real-world clinical lung cancer registry

**DOI:** 10.1007/s00508-024-02351-3

**Published:** 2024-04-23

**Authors:** Anna Lang-Stöberl, Hannah Fabikan, Maximilian Hochmair, Klaus Kirchbacher, Vania Mikaela Rodriguez, Leyla Ay, Christoph Weinlinger, David Rosenthaler, Oliver Illini, Nino Müser, Georg-Christian Funk, Arschang Valipour

**Affiliations:** 1 Karl Landsteiner Institute of Lung Research and Pulmonary Oncology, Klinik Floridsdorf and Klinik Ottakring, Vienna, Austria; 2 Department of Respiratory and Critical Care Medicine, Klinik Floridsdorf, Vienna Healthcare Group, Vienna, Austria; 3 2nd Department of Internal Medicine with Pneumology, Klinik Ottakring, Vienna Healthcare Group, Vienna, Austria

**Keywords:** NSCLC, SCLC, Database, NGS, Multicenter data collection

## Abstract

**Background:**

Lung cancer is a major health burden in Austria; however, limited real-world data exist on the diagnostic and treatment reality of lung cancer patients in Austria. The collection of high-quality data in a clinical setting is needed to gain insights into the real-world diagnostic and therapeutic management of lung cancer patients.

**Methods:**

The Karl Landsteiner Institute for Lung Research and Pulmonary Oncology implemented the Landsteiner lung cancer research platform (LALUCA), recruiting unselected lung cancer patients from two high volume centers in Vienna. This article describes the objectives, design, methodology of the registry and the process of implementation.

**Results:**

A multidisciplinary team of lung cancer specialists created a custom designed variable catalogue for the LALUCA platform consisting of 17 categories with 180 variables. Detailed information on clinical characteristics, diagnostic interventions, molecular pathology as well as curative and palliative treatment modalities are collected. During an implementation phase in 2020, the platform was optimized using the data of 50 patients. Since then a total of 1200 patients have been enrolled. Recruitment for the registry is ongoing with a recruitment rate of approximately 400 patients per year.

**Conclusion:**

The LALUCA registry is a web-based platform for the collection of real-world clinical data of lung cancer patients. Combining a large number of patients with a focus on gathering comprehensive data on diagnosis and treatment, the LALUCA registry provides a tool for investigation, evaluation, and improvement of the clinical management, survival and quality of care of Austrian lung cancer patients.

## Introduction

Lung cancer is the most common cause of cancer-related deaths and the second most frequently diagnosed cancer worldwide [1]. As in the rest of the world, lung cancer is a major health burden in Austria comprising 19% of all cancer deaths. With 2402 male and 1472 female deaths in 2017, it is the most frequent cause of cancer-related deaths in males and the second most frequent cause in females. Furthermore, the lung cancer incidence has shown a steady increase over the last five decades, comprising 11% of all newly diagnosed cancers [2]. Diagnosis and staging of lung cancer is a complex multistep process and the management of these patients requires a multidisciplinary and multimodal approach to determine the optimal treatment strategy, which includes surgery, radiotherapy, immunotherapy, chemotherapy and targeted approaches for tumors with driver mutations as well as individual combinations of all of the above [3].

The identification of driver mutations in a subset of tumors has altered the therapeutic landscape and substantially improved survival outcomes in lung cancer [4]. Therefore, efficient and effective molecular testing of lung cancer specimens is crucial. Due to the increasing number of molecular biomarkers, multiplexed assays such as next generation sequencing (NGS) are used to detect a multitude of drugable mutations in a single workflow [4].

Real-world data on routine practice of lung cancer management in Austria is scarce [2]. Gaining information on the diverse and complex population of lung cancer patients as a whole, however, is crucial as the enrolment of patients in clinical cancer trials has been shown to be highly selective including patients several years younger, with superior performance status and less comorbidities at the start of first line therapy than patients in the clinical setting [5–7]. Generalizability of recommendations based on clinical trials is therefore limited, as the results of clinical trials may reflect only outcomes under best-case scenarios. This results in a potential gap of information on patient outcomes especially among older patients or patients with comorbidities. The collection of real-world data complements the results from clinical trials by enabling us to gain insights into the incidence, characteristics and treatment reality of lung cancer patients and to improve healthcare policy decisions [7].

The Landsteiner lung cancer research platform (LALUCA) is a multicenter, clinical registry with prospective data obtained from lung cancer patients, with the aim of documenting data on molecular testing, diagnostic and treatment modalities as well as the course of disease. This article describes in detail the objectives, the methodology used and the implementation of the LALUCA registry.

## Patients, material and methods

### Population and registry area

The LALUCA registry is based in Vienna, the capital city of Austria. Vienna has a population of approximately 2 million inhabitants, who have free access to high-quality healthcare [8]. Between 2018 and 2020, 1028 cases of tracheal and lung cancer on average have been reported for Vienna to the Austrian National Cancer Registry [9]. The Departments of Respiratory and Critical Care Medicine of the Klinik Floridsdorf and the 2nd Department of Internal Medicine with Pulmonology of the Klinik Ottakring facilitate diagnosis and treatment for a large number of lung cancer patients in Vienna. Within this patient cohort, the LALUCA registry is recruiting patients for study participation.

### Objectives

The purpose of this project is to set up a clinical platform to collect representative real-world data on lung cancer management in Austria. We aim to evaluate clinical characteristics, diagnostic and treatment modalities, as well as outcome of unselected patients in real-life practice. In contrast to data of clinical trials, this registry is able to show data on the whole lung cancer patient population including information on older and comorbid patients. This registry focuses specifically on the collection of representative data on molecular biomarker testing and NGS data of patients before the start of first-line treatment, including information on rare mutations and clinical consequences of NGS testing. Another equally relevant objective of this registry is to assess indicators of the quality of care in patients with lung cancer such as timeliness of diagnosis and first treatment application as well as frequencies, modalities and sequences of imaging and treatment. In this respective, in an ongoing investigation we assess the implementation of biomarker testing by analyzing testing rates, timeliness and areas of improvement in molecular testing. By evaluating the incorporation of guideline recommendations, we hope to reveal challenges in molecular testing and highlight areas of improvement. Moreover, investigations of specific therapeutic settings are planned to analyze outcomes in a real-world patient cohort, thereby complementing results from cancer trials and adding information for the development of guidelines and policy decisions.

### Study design

The LALUCA registry is registered at ClinicalTrials.gov (NCT04733430) as an observational, prospective, multicenter clinical registry. Overseen by the Karl Landsteiner Institute for Lung Research and Pulmonary Oncology, the LALUCA registry is run by a multidisciplinary team, including pulmonologists, pathologists, oncologists, thoracic surgeons, radio-oncologists, clinical scientists, system engineers and several data entry specialists. The registry is designed as a custom web-based platform created by celsius37^TM^ (celsius37.com AG, Mannheim, Germany) using the push method of data extraction and input by trained staff. Data entry personnel (DEP), each working with a separate user account, extract detailed information from electronic health records (EHR) and transform running text into specific variables that are manually documented into standardized electronic case report forms (eCRF). The eCRF is filled out on the LALUCA website in accordance with good clinical practice (GCP) guidelines, as well as with additionally created LALUCA guidelines ensuring the accuracy, legibility, completeness and timeliness of data documentation.

### Recruitment

All patients, who are diagnosed with lung cancer at one of the two centers, are offered participation. Patients are eligible for inclusion if they have a histologically or cytologically confirmed lung cancer, are over the age of 18 years, and sign a consent form. Additionally, patients need to be able to understand and complete the patient reported outcomes assessment instruments. Recruitment for patients started in November 2020 and is ongoing.

### Ethics and sponsors

The study was reviewed and approved by the ethics committee of Vienna (EK-20-061-VK). This project is supported by the following industry partners: Amgen, AstraZeneca, Bristol Myers Squibb, Böhringer, Eli Lilly Oncology, Gilead, Janssen, Merck Group, Merk Sharp&Dohme, Novartis, Pfizer, Roche, Sanofi, and Takeda Oncology. Sponsors had no influence on design, methodology, or parameter selection of the registry.

### Data collection and measurements

After patients have signed the informed consent form, the LALUCA team is given access to their medical health records relevant to the management of lung cancer. Additionally, patients are required to fill out a case history questionnaire. To enable DEP to gather all relevant information for the eCRF in the LALUCA registry, specifically tailored forms are available to the physicians in either digital or physical form for patient documentation. The collection of patient information starts at the first consultation and includes the patient’s self-reported questionnaires, tailored forms from the physicians, as well as imaging records, pathological, histological and cytological reports from the EHR. All obtained data variables organized by category are shown in the Table 1. As an example for the documentation of specific elements on the platform, collected data items for molecular pathology are displayed in Table 2.

**Table 1:** Data categories and variables of the LALUCA registry. *ECOG* eastern cooperative oncology group, *FEV* forced expiratory volume, *DLCO* diffusion capacity for carbon monoxide, *FVC* forced vital capacity, *pTNM* pathological tumor-node-metastasis, *PD-L1* programmed death-ligand 1, *TPS* tumor proportion score, *IHC* immunhistochemistry, *PCR* polymerase chain reaction, *FISH* fluorescence in situ hybridization, *NGS* next generation sequencing, *EGFR* epidermal growth factor receptor, *VAMLA*,video-assisted mediastinoscopic lymphadenectomy, *IP* investigational product

Category	Variables	
Demographics and anthropometrics	Gender, year of birth, ethnicity, weight (kg), height (cm), patient’s profession	
Basic clinical information	Presentation mode (screening, symptoms, incidental findings), basis of diagnosis (clinical, histological, cytological), first clinical visit, diagnosis on site, referral, previous treatments	
Further clinical information	First symptoms	–
	ECOG status	At diagnosis
	Lung function test at baseline	Date, FEV1, FEV1%, DLCO%, FVC, FVC%
	Laboratory tests	–
Smoking history	Smoking status	Status, pack years, start year and stop year
Medical history	Comorbidities	Type and severity of disease
Radiological diagnostics	CT scan PET-CT scan Sonography Scintigraphy Others	Date and location
Invasive diagnostics	Bronchoscopy Diagnostic surgery CT-guided biopsy Ultrasound guided biopsy Surgical resection of metastases	Date and location
Pathological diagnostics	Date of biopsy, biopsy location, pTNM stage, perineural invasion, invasion of visceral pleura, vein invasion, lymphatic invasion, resection grade, PD-L1 (date, PD-L1 TPS score)	
	Histological subtype	Subtype and subclassification
	Biomarker:	Biomarker, date of report & testing method (IHC/PCR/FISH/NGS)
Molecular diagnostics—NGS	Next-generation sequencing, date of sampling, date of results, location of biopsy, DNA sequencing pathogens/target mutation, RNA sequencing pathogen/target mutation fusion, Copy number changes pathogens/target	
Liquid biopsy	Method, date of blood collection, date of pathology report, reason, results, other mutation, EGFR gene copy number	
Staging & follow-up	Visit type and date, localization and side of primary tumor, radiological diagnostic method, tumor board, stadium, T, N, M stage, staging system, visit reason, metastasis location, new symptoms, response assessment, progression type, lesion of progression, follow-up consequences	
Curative treatment	Type and name of treatment, start and end date, adverse events, treatment interruption or modification, number of cycles, regimen	
Palliative treatment	Type and name of treatment, start and end date, adverse events, treatment interruption or modification, number of cycles, regimen	
Radiation therapy	Type of treatment, location, intent, date, dose per fraction, number of fractions, reason for discontinuation, regimen	
Operation and invasive therapy	Type of therapy, date, surgical removal, pleural intervention, operation type, operation location, surgery type, VAMLA	
Survival follow-up	Patient status, reason of death, date of death, last date known alive	
Study participation	Clinical study participation, name of study, IP, start and end date

**Table 2:** Example of the complete documentation of biomarker diagnostics. *PD-L1* programmed death ligand 1, *ALK* anaplastic lymphoma kinase gene, *ROS1* ros proto-oncogene 1, *BRAF* b-raf proto-oncogene, *EGFR* epidermal growth factor receptor, *MET* MET proto-oncogene, *RET* ret proto-oncogene, *NTRK* neurotrophic tropomyosin receptor kinase, *KRAS* Kristen rat sarcoma virus, *HER2* human epidermal growth factor receptor

Examples of specific elements		
*Pathological diagnostics*		
Date of biopsy	*Dd.mm.yyyy*	
Date of results	*Dd.mm.yyyy*	
Location of biopsy	*Text*	
PD-L1	PD-L1 TPS score (%) PD-L1 date	
ALK fusion	Positive Negative Failed/unclear/not done	
ROS1 fusion	Positive Negative Failed/unclear/not done	
BRAF mutation	V600E/D/D2 Exon 11 Other Negative Failed/unclear/not done	
EGFR mutation	Exon 19 deletion Exon 21(L858R) Exon 21(L861Q) G719x T790m S768i Exon 20 insertion Other Negative Failed/unclear/not done	
MET mutation	MET exon 14 skipping mutation Other MET exon 14 mutation Other MET mutation Positive Negative Failed/unclear/not done	
MET amplification	Low High Negative Failed analysis/unclear/not done	Gene copy Number < 10 Gene copy Number ≥ 10
RET fusion	Positive Negative Failed/unclear/not done	
NTRK fusion	NTRK1 NTRK2 NTRK3 Negative Failed/unclear/not done	
KRAS mutation	G12c Other Negative Failed/unclear/not done	
HER2 neu amplification (ERBB2)	Positive Negative Failed/unclear/not done	Gene copy Number > 0 Gene copy Number = 0
HER2 neu mutation (ERBB2)	Positive Negative Failed/unclear/not done	Gene copy Number > 0 Gene copy Number = 0

### Data quality control

To guarantee a sufficiently high quality of the data, various measures were implemented to ensure data accuracy, completeness, capture, data standardization and timeliness [10]. First, all DEP are trained by senior staff members and participate in a monthly training seminar to update their knowledge and to be equipped with all tools necessary to abstract relevant information from the EHR and enter it into the eCRFs. To enhance data accuracy and to reduce the possibility of interpretative errors, the LALUCA guidelines were created containing all the data items, codes and definitions assessed in the registry and therefore serve as a data dictionary during the input process. All DEP have access to the LALUCA guidelines to allow a uniform interpretation of EHR and input into the data registry. Second, the tailored forms filled out by physicians serve the purpose of inputting complete information into the LALUCA registry. Third, input variables are mostly standardized and seldom allow free text. Finally, quality assurance monitoring is carried out by a clinical monitor throughout the whole study and data quality checks are performed periodically using custom-made Python scripts including completeness and extensive plausibility checks, as well as regular statistical reports of the data sets. In cases of discrepancies or missing values, the study sites are contacted to make any necessary changes to the data.

### Follow-up

After enrolment, patient data are assessed every 6 months for a follow-up status and an update of ongoing treatment. In cases of loss to follow-up, the insurance provider is contacted for information about the patient’s survival status.

## Results

### Implementation

The development and implementation of the LALUCA registry is an effort of the Karl Landsteiner Institute for Lung Research and Pulmonary Oncology. The need for a high-quality clinical registry, especially with respect to the collection of representative real-world data on molecular biomarkers in unselected patients, was recognized and a prospective multicenter clinical registry developed. A catalogue consisting of 17 categories with 180 variables was collected based on numerous discussion rounds with specialists from pulmonology, pathology, oncology, thoracic surgery and radio-oncology. After the implementation of a web-based platform according to the custom designed variable catalogue in the first phase of the project, a test run of the system was conducted allowing system optimization and a successful launch of the registry.

### Growing participation

**Fig. 1:**
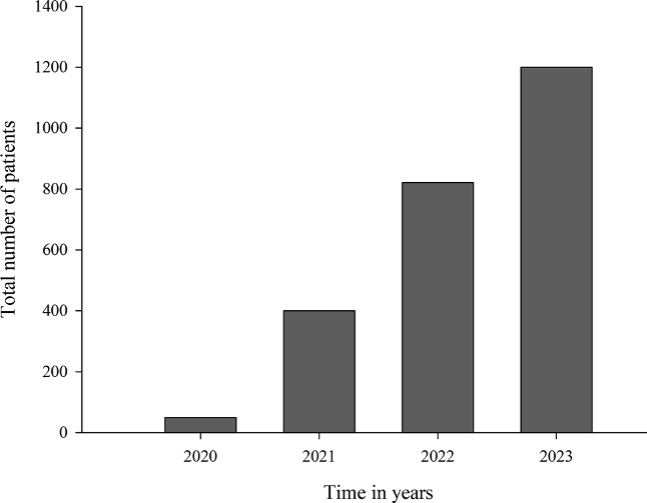
Evolution of the patient participation in the LALUCA registry

During the implementation phase of the registry from November 2020 to December 2020, 50 patients were included. The total number of patients increased rapidly over time and the project is ongoing with a recruitment rate of about 400 patients annually. Patient enrolment across both research sites is displayed in Fig. 1. In 2023 the number of patients included in the registry rose to a total of 1200 patients, who are regularly followed up creating ever growing real-world data.

### Preliminary results

**Fig. 2:**
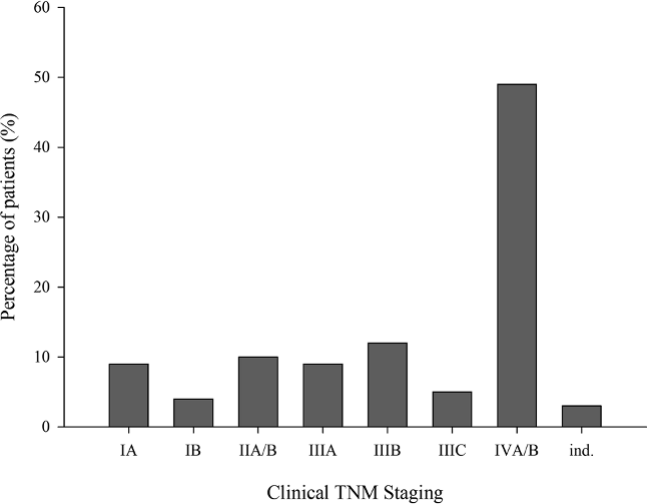
Staging at initial diagnosis. *ind.* indetermined, *TNM* tumor-node-metastasis

Of 1200 patients included in the registry, 159 patients were excluded from the analysis due to the findings of either synchronous primary lung carcinoma or the tumor being a recurrence, 46% of the patients were female and 54% male. The majority of patients (70%) were 61–80 years old and either current (51%) or former smokers (38%). With 49%, most patients were diagnosed at stage IVA/B (Fig. 2).

In the registry adenocarcinoma was the most prevalent histological subtype with 54.3%, followed by squamous cell carcinoma with 20.5%. The prevalence of all histologic subtypes is shown in Table 3.

**Table 3:** Prevalence of histological subtypes

Pathological diagnostics	
Histological subtypes	%
Adenocarcinoma	54.3
Squamous cell carcinoma	20.5
Small cell lung cancer	14.1
NSCLC—not otherwise specified	9.0
Large cell neuroendocrine carcinoma	0.9
Other neuroendocrine tumor	0.8
Adenosquamous carcinoma	0.3
Sarcomatoid carcinoma	0.1

### Project continuation and outlook

The end of 2023 not only marks the inclusion of a total of 1200 patients in the LALUCA registry but also the transition to an improved version with updated variables, upgraded eCRFs and data transfer to an improved IT infrastructure. We used what we have learned so far to improve and simplify data input and management by further standardization and user-friendly drop-down options. Furthermore, to accommodate the increasing load of data, additional positions for DEP were created. Additionally, collaborations with other Austrian sites are in the planning stage.

## Discussion

The LALUCA registry continuously recruits unselected lung cancer patients from two high volume sites in Vienna, Austria, since its implementation in 2020. Using a custom web-based platform, comprehensive and detailed information on molecular testing, diagnostic and treatment modalities as well as the course of disease is collected in a real-world clinical dataset.

To date, several registries have been implemented to record and evaluate the burden of cancer in the Austrian population. On a national level, the Austrian national cancer registry serves as a data collection tool for epidemiological purposes. It collects data on incidence, prevalence, mortality, and survival probability of all types of cancer based on the entire population of Austria since its implementation in 1983 [11]. Following the development of the Austrian national cancer registry, regional registries have emerged in Carinthia, Salzburg, Tyrol and Vorarlberg, which collect and provide epidemiological data for the national cancer registry, while also using their data to improve the quality of care in their Federal State [12–15]. In recent years, hospital-based clinical quality data registers have evolved in Lower and Upper Austria, pooling data from 24 and 11 hospitals, respectively. As Lower and Upper Austria are the Federal States with the second and third highest population in Austria, these registries gather data from areas populated by 1.7 and 1.5 million inhabitants, respectively [8]. Thus, both registries, combining web-based software tools with teams for quality management, provide detailed and comprehensive data on cancer patients in their respective Federal State [16, 17]. The Federal State of Vienna encompasses a population as large as 2 million inhabitants [8]. Between 2018 and 2020 the incidence rates of tracheal and lung cancer were the highest in Vienna with a total of 1028 cases compared to 964 and 728 cases in Lower and Upper Austria, respectively [9]. Within this patient collective, the LALUCA registry currently includes approximately 400 new patients on a yearly basis.

The LALUCA registry stands out among national and international lung cancer registries. In accordance with the current national guidelines all NSCLC patients in the LALUCA registry undergo reflex testing with an extended NGS biomarker panel; however, internationally the practice of reflex testing has been reported in only 14 of 21 European countries [18, 19]. Moreover, only a third of European laboratories use single gene testing for epidermal growth factor receptor (EGFR) mutations followed by further testing with NGS in only 45% of cases [20]. The LALUCA registry offers real-world NGS data on a wide variety of histological subtypes including squamous carcinomas and small cell lung cancer and collects crucial data for the assessment of molecular biomarkers. Furthermore, the registry has an extensive collection of surgical and oncologic treatments, which also allows the investigation of clinical consequences of NGS testing on therapeutic concepts. To the best of our knowledge, these advantages set the LALUCA registry apart from other international registries such as the Danish lung cancer registry, which cannot provide information on curative or palliative treatment intent or specify oncological treatment, or the National Lung Cancer Audit UK, which only requires EGFR mutation status, ALK and ROS1 fusion status and PD-L1 expression for minimal completion of a patient thereby limiting detailed information on molecular biomarkers in their overall dataset [21–23]. Additionally, our platform facilitates the collection of key data on specific supportive therapies and the investigation on changes in the diagnostic and therapeutic approach over the course of time in all types and stages of lung cancer in contrast to other international registries which only record the planned primary treatment such as the Swedish National Lung Cancer Register or only include advanced NSCLC patients such as the German CRISP registry [23, 24]. Future directions of our registry include investigations of incorporation of guideline recommendations regarding molecular testing for the assessment of quality of care as well as investigations of real-world patient outcomes in specific therapeutic settings for the complementation of trial results with data from a diverse and complex patient population.

The design of the LALUCA registry has some clear strengths: first, a multidisciplinary team of clinicians was involved in establishing the registry design and determining variables included in the dataset. The engagement of clinicians has shown improvement in the quality and usefulness of cancer registry data by ensuring the collection of relevant variables [26]. Due to the fast changes in the field of oncology, the clinicians’ perspective and a close interdisciplinary collaboration are imperative to understand which data variables are required to assess new clinical practices and to shed light on current treatment paradigms [25, 26]. Second, data entry in the LALUCA registry is performed by specially trained DEP using a data dictionary, specially curated by clinicians, for uniform interpretation of definitions and standardized input of data. Standardization and clear definition of variables reduces high interobserver variability of DEP that has been found to be an issue in other registries [27]. Especially, the involvement of clinicians in the development of data dictionaries for data standardization has been recommended to establish clear data definitions and thereby quality data [25]. Third, in the rapidly progressing field of oncology, the importance for registries to remain flexible and adaptive in order to stay relevant has been demonstrated [26, 28]. All variables included in the registry need to be carefully evaluated in order to be relevant and useful, as too many variables result in an increased workload of text mining and data entry for the registry team without adding additional benefit [28]. Accordingly, the LALUCA registry was updated to include re-evaluated variables, adding the newly relevant while omitting the obsolete. Furthermore, high data quality of the LALUCA registry is guaranteed by quality assurance monitoring and data quality checks which involve the detection and correction of missing or erroneous data.

A limitation of our registry, however, is that while the LALUCA registry recruits a representative sample of lung cancer patients, it does not cover the majority of patients diagnosed with lung cancer in the Federal State of Vienna, yet. The Steering Committee of the registry, though, has already reached out to other high volume lung cancer sites in Vienna and other cities, to further expand data collection.

In conclusion, the LALUCA registry has been designed to create high quality clinical data on lung cancer. With its primary focus on comprehensive NGS data and its high number of patients, it offers an important platform to investigate real-world practice and to evaluate the quality of care for patients with lung cancer.

## Abbreviations

DEP: Data entry personnel
eCRF: Electronic case report file
EGFR: Epidermal growth factor receptor
EHR: Electronic health records
GCP: Good clinical practice
LALUCA: Landsteiner lung cancer research platform
NGS: Next generation sequencing
NSCLC: Non-small cell lung cancer

## References

[1] Sung H, Ferlay J, Siegel RL, Laversanne M, Soerjomataram I, Jemal A, et al. Global Cancer Statistics 2020: GLOBOCAN Estimates of Incidence and Mortality Worldwide for 36 Cancers in 185 Countries. CA Cancer J Clin. 2021;71(3):209–49. 33538338 10.3322/caac.21660

[2] Pirker R, Prosch H, Popper H, Klepetko W, Dieckmann K, Burghuber OC, et al. Lung Cancer in Austria. J Thorac Oncol. 2021;16(5):725–33. 33896572 10.1016/j.jtho.2020.10.158

[3] Batra U, Munshi A, Kabra V, Momi G. Relevance of multi-disciplinary team approach in diagnosis and management of Stage III NSCLC. Indian J Cancer. 2022;59(5):46. 35343190 10.4103/ijc.IJC_51_21

[4] Tan AC, Tan DSW. Targeted Therapies for Lung Cancer Patients With Oncogenic Driver Molecular Alterations. J Clin Oncol Off J Am Soc Clin Oncol. 2022;40(6):611–25. 10.1200/JCO.21.01626 34985916

[5] von Verschuer U, Schnell R, Tessen HW, Eggert J, Binninger A, Spring L, et al. Treatment, outcome and quality of life of 1239 patients with advanced non-small cell lung cancer—final results from the prospective German TLK cohort study. Lung. Cancer. 2017;112:216–24. 10.1016/j.lungcan.2017.07.031 28916198

[6] Murthy VH, Krumholz HM, Gross CP. Participation in Cancer Clinical Trials: Race‑, Sex-, and Age-Based Disparities. JAMA. 2004;291(22):2720. 10.1001/jama.291.22.2720 15187053

[7] Penberthy LT, Rivera DR, Lund JL, Bruno MA, Meyer A. An overview of real-world data sources for oncology and considerations for research. CA Cancer J Clin. 2022;72(3):287–300. 34964981 10.3322/caac.21714

[8] Statistik Austria. Data from Austria’s federal institution „Statistik Austria“. https://www.statistik.at/statistiken/bevoelkerung-und-soziales/bevoelkerung/bevoelkerungsstand/bevoelkerung-zu-jahres-/-quartalsanfang. Accessed Nov 15, 2023.

[9] Statistik Austria. Data from Austria’s federal institution „Statistik Austria“: Krebsinzidenz nach ausgewählter Lokalisation, Bundesland und Geschlecht, Jahresdurchschnitt (2018–2020). https://www.statistik.at/statistiken/bevoelkerung-und-soziales/gesundheit/krebserkrankungen/. Accessed November 15, 2023.

[10] Prang KH, Karanatsios B, Verbunt E, Wong HL, Yeung J, Kelaher M, et al. Clinical registries data quality attributes to support registry-based randomised controlled trials: A scoping review. Contemp Clin Trials. 2022;119:106843. 35792338 10.1016/j.cct.2022.106843

[11] Statistik Austria. (Definition, Erläuterung, Methoden, Qualität) zur Krebsstatistik/Krebsregister. Bundesanstalt Statistik Österreich; 2023.

[12] Tumorregister Tirol (TRT).. https://www.iet.at/page.cfm?vpath=register/tumorregister/. Accessed 11 Nov 2023.

[13] Epidemiologie von Krebserkrankungen. Österreichischer Krebsreport.. https://www.krebsreport.at/2021/epidemiologie/epidemiologie-von-krebserkrankungen/. Accessed 15 Nov 2023.

[14] Krebsregister Vorarlberg (Cancer Registry Vorarlberg).. https://www.aks.or.at/science/cancer-registry/. Accessed 15 Nov 2023.

[15] Tumorregister des Bundeslandes Salzburg.. https://onkologie-salzburg.com/tumorregister/. Accessed 15 Nov 2023.

[16] Qualitas Redaktion. Das NÖ Onkologie-Informationssystem. CGM Global. 6. November 2021;

[17] MMag. Sigrid Metz-Gercek. Smart-Data anstelle von Big Data – Erfolgskriterien für Krebsregister. 25. February 2021;Spectrum Onkologie.

[18] Popper HH, Gruber-Mösenbacher U, Pall G, Müllauer L, Hochmair M, Krenbek D, u. a. The 2020 update of the recommendations of the Austrian working group on lung pathology and oncology for the diagnostic workup of non-small cell lung cancer with focus on predictive biomarkers. Memo—Mag Eur Med Oncol. 2020;13(1):11–26.

[19] Thunnissen E, Weynand B, Udovicic-Gagula D, Brcic L, Szolkowska M, Hofman P, et al. Lung cancer biomarker testing: perspective from Europe. Transl Lung Cancer Res. 2020;9(3):887–97. 32676354 10.21037/tlcr.2020.04.07 PMC7354119

[20] Hofman P, Calabrese F, Kern I, Adam J, Alarcão A, Alborelli I, et al. Real-world EGFR testing practices for non-small-cell lung cancer by thoracic pathology laboratories across Europe. Esmo Open. 2023;8(5):101628. 37713929 10.1016/j.esmoop.2023.101628 PMC10594022

[21] COSD key data items for collection 2022. 2023. https://www.lungcanceraudit.org.uk/content/uploads/2022/04/NLCA_key_data_items_April-2022.pdf. Accessed 10 Jan 2024.

[22] Rich AL, Baldwin DR, Beckett P, Berghmans T, Boyd J, Faivre-Finn C, et al. ERS statement on harmonised standards for lung cancer registration and lung cancer services in Europe. Eur Respir J. 2018;52(6):1800610. 30361252 10.1183/13993003.00610-2018

[23] Gouliaev A, Rasmussen TR, Malila N, Fjellbirkeland L, Löfling L, Jakobsen E, u. a. Lung cancer registries in Denmark, Finland, Norway and Sweden: a comparison and proposal for harmonization. Acta Oncol. 2. January 2023;62(1):1–7. 10.1080/0284186X.2023.2172687 36718556

[24] Griesinger F, Eberhardt W, Nusch A, Reiser M, Zahn MO, Maintz C, u. a. Biomarker testing in non-small cell lung cancer in routine care: Analysis of the first 3,717 patients in the German prospective, observational, nation-wide CRISP Registry (AIO-TRK-0315). Lung Cancer. 2021;152:174–84. 10.1016/j.lungcan.2020.10.012 33358484

[25] Asare EA, Gress DM, Greene FL, Winchester DP. Cancer registry data: Engaging the clinician to improve quality. Cancer. 2015;121(17):2866–7. 26018328 10.1002/cncr.29483

[26] MacIntyre M, MacKay C. Lessons learned from the Canadian cancer registry experience. Healthc Manage Forum. 2018;31(1):9–12. 29264973 10.1177/0840470417733008

[27] Platell C, Penter C. Cancer auditing—how accurate are your data? Colorectal Dis. 2013;15(2):164–8. 22731686 10.1111/j.1463-1318.2012.03143.x

[28] Wormald JS, Oberai T, Branford-White H, Johnson LJ. Design and establishment of a cancer registry: a literature review. ANZ J Surg. 2020;90(7–8):1277–82. 10.1111/ans.16084 32564454

